# Antithrombotic Therapy After Intracerebral Hemorrhage: Real‐World Evidence for In‐Hospital Resumption

**DOI:** 10.1002/cns.70883

**Published:** 2026-04-28

**Authors:** Shengde Li, Tian Qu, Xiang Zhou, Qi Miao, Jun Ni, Bin Peng

**Affiliations:** ^1^ Department of Neurology Peking Union Medical College Hospital, Chinese Academy of Medical Science and Peking Union Medical College Beijing China; ^2^ Department of Information Center Peking Union Medical College Hospital, Chinese Academy of Medical Science and Peking Union Medical College Beijing China; ^3^ Department of Cardiac Surgery Peking Union Medical College Hospital, Chinese Academy of Medical Science and Peking Union Medical College Beijing China

**Keywords:** intracerebral hemorrhage, ischemic stroke, prognosis, restart of antithrombotic therapy

## Abstract

**Background and Purpose:**

Patients with intracerebral hemorrhage (ICH) who require antithrombotic therapy (AT) face competing risks of recurrent bleeding and ischemic events. Optimal timing of AT resumption during hospitalization remains uncertain. We evaluated whether restarting AT in hospitalized patients with acute spontaneous ICH reduces ischemic complications without increasing hemorrhagic risk.

**Methods:**

We conducted a retrospective cohort study at Peking Union Medical College Hospital (2014–2022) including adults (≥ 18 years) admitted with spontaneous ICH within 6 months of onset. Patients were categorized as: Group 1, no AT indication; Group 2, AT indicated but not treated; Group 3, AT indicated and treated. Primary endpoints were ischemic events (ischemic stroke, transient ischemic attack, myocardial infarction, pulmonary embolism, deep venous thrombosis) and hemorrhagic events (new ICH or hematoma expansion ≥ 12.5 mL or ≥ 33%). Outcomes were assessed from onset to discharge.

**Results:**

Among 601 patients (median age 58 years; 40.3% female), 256 (42.6%) were in Group 1, 247 (41.1%) in Group 2, and 98 (16.3%) in Group 3. Median time from onset to AT resumption was 11 days (IQR 2–27). Restarting AT (Group 3) significantly reduced ischemic events (14.3% vs. 28.7%; adjusted HR 0.34, 95% CI 0.18–0.65) without increasing hemorrhagic events (5.1% vs. 6.3%; adjusted HR 0.67, 95% CI 0.20–2.19). Competing‐risk models and sensitivity analysis confirmed these findings.

**Conclusions:**

In‐hospital resumption of AT after acute spontaneous ICH significantly decreased ischemic events without excess hemorrhagic risk, supporting its potential benefit in carefully selected patients.

## Graphical abstract

In‐hospital resumption of antithrombotic therapy after acute spontaneous intracerebral hemorrhage was associated with a reduced risk of ischemic events and no excessive bleeding risk.

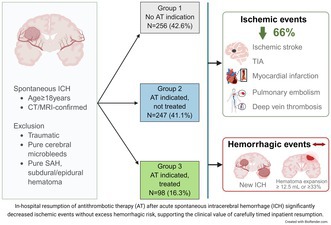

## 1 Introduction

Intracerebral hemorrhage (ICH) is one of the most disabling and fatal forms of stroke, affecting over 4.5 million individuals annually [1]. Acute spontaneous intraparenchymal hemorrhage accounts for the majority of ICH cases and carries both a high risk of recurrence and a substantial risk of subsequent ischemic events [2, 3]. Many survivors of ICH require antithrombotic therapy (AT) for coexisting conditions such as atrial fibrillation, prior ischemic stroke, or venous thromboembolism. Resuming AT after ICH represents a critical clinical dilemma: balancing prevention of ischemic complications against the danger of recurrent bleeding. Although guideline recommendations support restarting AT when clearly indicated, clinical practice is highly variable. Up to 82% of clinicians report an intention to resume anticoagulation after ICH, yet fewer than 20% implement it in the early post‐ICH period [4, 5].

Existing studies, including RESTART, suggest that AT resumption can be safe and beneficial under selected conditions [6, 7, 8, 9]. However, most available data are derived from atrial fibrillation cohorts and focus on post‐discharge initiation, often weeks to months after the index event [6, 10]. Moreover, the efficacy of AT after ICH remained controversial, and the decision‐making is quite variable in practice [7, 10]. This leaves a critical gap regarding whether restarting AT during hospitalization for acute spontaneous ICH can provide net clinical benefit.

To capture real‐world decision‐making, our study included patients admitted within 6 months of ICH onset, encompassing both the acute phase (≤ 14 days) and later presentations. This design reflected the clinical reality that some patients with severe ICH require prolonged hospitalization beyond 2 weeks for intensive management, rehabilitation, or delayed transfer, making the timing of AT resumption during extended inpatient care clinically relevant [11, 12].

We hypothesized that restarting AT during hospitalization in patients with acute spontaneous ICH reduces ischemic complications without increasing recurrent hemorrhage.

## 2 Methods

### 2.1 Study Population

Restart‐R was a single‐center, retrospective cohort study conducted at Peking Union Medical College Hospital from November 2014 to October 2022. We included consecutive adult patients (≥ 18 years) hospitalized with acute spontaneous intraparenchymal ICH, confirmed by CT or MRI, who were either admitted within 6 months (≤ 180 days) of symptom onset or experienced in‐hospital ICH. Exclusion criteria were as follows: (1) Traumatic ICH; (2) Pure cerebral microbleeds on susceptibility‐weighted imaging; (3) Pure subarachnoid hemorrhage, subdural hematoma, epidural hematoma. This study was approved by the Ethics Committee of Peking Union Medical College Hospital, with a waiver of informed consent (I‐22PJ776).

### 2.2 Data Collection

Baseline data included demographics, vascular risk factors, medical history, vital signs, and laboratory values. Stroke severity and functional status were assessed per standard guidelines using the National Institutes of Health Stroke Scale (NIHSS), modified Rankin Scale (mRS), Glasgow Coma Scale (GCS), ICH score, HAS‐BLED, Essen Stroke Risk Score (ESRS), and CHA_2_DS_2_‐VASc [13, 14, 15, 16]. Hematoma characteristics were recorded, including location, laterality, and volume calculated by the ABC/2 method. ICH etiology was classified using the modified SMASH‐U criteria [17]. All assessments were confirmed by an independent senior neurologist panel led by Bin Peng.

### 2.3 Antithrombotic Therapy Exposure

Patients were stratified into three groups: Group 1: No indication for antithrombotic therapy; Group 2: Indication for AT but no antithrombotic therapy restarted during hospitalization; Group 3: Indication for AT and antithrombotic therapy restarted during hospitalization. Antithrombotic agents were categorized as antiplatelet, anticoagulant, or combination therapy. Timing of AT resumption was defined as the interval (days) from ICH onset to the first AT dose. Admission window (≤ 14 days vs. 15–180 days) was recorded for prespecified subgroup analysis.

### 2.4 Outcomes

Primary efficacy endpoint: ischemic events (ischemic stroke, transient ischemic attack, myocardial infarction, pulmonary embolism, deep vein thrombosis). Primary safety endpoint: recurrent ICH or hematoma expansion, defined as an absolute increase ≥ 12.5 mL or a relative increase ≥ 33% from baseline volume. Secondary outcomes: any intracerebral hemorrhage, extracranial bleeding (gastrointestinal, urinary, ocular), favorable functional outcome (mRS ≤ 3 at discharge), GCS ≥ 9 at discharge, all‐cause mortality, vascular mortality, and discharge against medical advice (DAMA). DAMA was included as a secondary outcome because, in our setting, a proportion of critically ill ICH patients with poor prognosis are withdrawn from active treatment and discharged at family request, which may confound mortality estimates [18].

All outcome events were adjudicated by a blinded, independent committee. In Group 3, events were further analyzed relative to the timing of AT initiation (pre‐ vs. post‐restart).

### 2.5 Etiology Analysis of Recurrent ICH and Death

An independent, blinded committee (led by Jun Ni) analyzed recurrent ICH causes and death cases based on medical records.

### 2.6 Follow‐Up Time

Follow‐up data, including GCS and mRS scores, complications, and clinical events, were collected from onset to hospital discharge.

### 2.7 Statistical Analysis

Continuous variables with non‐normal distributions were expressed as medians (interquratile ranges[IQR]). Categorical variables were presented as frequencies and percentages. Group comparisons were conducted using Wilcoxon rank‐sum, Kruskal‐Wallis, or *χ* ^2^ tests as appropriate. Cumulative incidence curves were used to depict clinical outcomes across the groups. Cox proportional hazards models were employed to assess associations with ischemic/hemorrhagic events, intracerebral hemorrhage, extracranial bleeding, and death. For ischemic event, hemorrhagic event, any intracerebral hemorrhage, and extracranial bleeding, Fine–Gray sub‐distribution hazard models were used to account for the competing risk of death and were presented as sub‐distribution hazard ratios [19]. Logistic regression models were applied for mRS ≤ 3 and GCS ≥ 9 at discharge. A complete unadjusted Cox or Logistic regression with all baseline variables was performed initially, followed by adjustments for potential confounders.

Subgroup analyses explored whether AT's association with ischemic/hemorrhagic events varied by age (≤ 49 years vs. ≥ 50 years), gender, ICH score (≤ 3 vs. ≥ 4), HAS‐BLED score (≤ 2 vs. ≥ 3), etiology (hypertension vs. non‐hypertension) and time from onset to admission (≤ 14 days vs. 15–180 days). Sensitivity analyses assessed the robustness of these associations. Among patients with AT indications (Groups 2 and 3), outcomes related to specific antithrombotic drugs (antiplatelet vs. anticoagulant) were tested. Cox or logistic models were adjusted for baseline hemorrhagic lesion size (< 5 mL vs. ≥ 5 mL). Missing data were deleted from analysis.

Statistical analyses were conducted using the SAS 9.3 (SAS Institute Inc.). All tests were two‐sided and *p* values < 0.05 were considered statistical significance.

## 3 Results

### 3.1 Patient Characteristics

Among 2631 screened patients with intracerebral hemorrhage, 601 met the inclusion criteria of acute spontaneous intraparenchymal ICH and were admitted within 6 months of onset or developed in‐hospital ICH (Figure S1). The median age at onset was 58 years (IQR:46‐69 years), and 242 patients were female (40.3%). Of these, 256 patients (42.6%) had no indication for antithrombotic therapy (AT), 247 (41.1%) had an indication but did not receive AT, and 98 (16.3%) had an indication and were restarted on AT during hospitalization.

Baseline characteristics are summarized in Table 1. In brief, patients receiving AT were more likely to have a history of ischemic stroke or atrial fibrillation, had smaller median hematoma volumes (1.9 mL vs. 17.0–17.9 mL) and lower ICH scores at admission, and less frequently required craniotomy. Rate of intraventricular hemorrhage was lower in patients receiving AT (15.3%). Core clinical features and acute management are shown in Table 2; extended laboratory and radiologic data are provided in Table S1. Median follow‐up was 21 (11–36), 20 (7–38), and 36 (24–62) days in Groups 1–3, respectively (*p* < 0.0001).

**TABLE 1:** Demographic and medical history of patients with intracerebral hemorrhage.

	Group 1, *N* = 256	Group 2, *N* = 247	Group 3, *N* = 98	*p*
Age, years	52 (40‐64)	63 (52‐72)	62 (48‐72)	< 0.0001
Sex, female	100 (39.1)	102 (41.3)	40 (40.8)	0.8714
Minority	12 (4.7)	11 (4.5)	2 (2.0)	0.5127
Hypertension	121 (47.3)	153 (61.9)	50 (51.0)	0.0035
Diabetes mellitus	30 (11.7)	71 (28.7)	24 (24.5)	< 0.0001
Dyslipidemia	14 (5.5)	34 (13.8)	10 (10.2)	0.0069
History of stroke	22 (8.6)	97 (39.3)	57 (58.2)	< 0.0001
History of IS	0 (0.0)	93 (37.7)	54 (55.1)	< 0.0001
History of HS/SAH	22 (8.6)	9 (3.6)	5 (5.1)	0.0598
History of HD	6 (2.3)	84 (34.0)	38 (38.8)	< 0.0001
AF	0 (0.0)	21 (8.5)	15 (15.3)	< 0.0001
Autoimmune disease	19 (7.4)	25 (10.1)	14 (14.3)	0.1397
Thrombocytopenia	36 (14.1)	44 (17.8)	7 (7.1)	0.0385
CKD	6 (2.3)	21 (8.5)	2 (2.0)	0.0021
Renal dialysis	0 (0.0)	15 (6.1)	1 (1.0)	< 0.0001
Malignant tumor	73 (28.5)	68 (27.5)	12 (12.2)	0.0044
Smoking, ever	53 (20.7)	77 (31.2)	24 (24.5)	0.0258
Regular drinking, ever	36 (14.1)	52 (21.1)	20 (20.4)	0.0983
Obesity^a^	33 (15.8)	22 (11.4)	9 (10.8)	0.3375
Family history of cardio‐vascular disease^b^	26 (10.2)	33 (13.4)	15 (15.3)	0.3384
Family history of HS^c^	12 (4.7)	15 (6.1)	7 (7.1)	0.6262
Extracranial bleeding^d^	29 (11.3)	44 (17.8)	5 (5.1)	0.0039
Prior surgery^e^	4 (1.6)	30 (12.1)	14 (14.3)	< 0.0001
Cardiovascular	1 (0.4)	23 (9.3)	12 (12.2)	< 0.0001
Non‐cardiovascular	3 (1.2)	7 (2.8)	2 (2.0)	0.4115
Use of antiplatelet agents^f^	13 (5.1)	82 (33.2)	38 (38.8)	< 0.0001
Use of anticoagulants^g^	3 (1.2)	61 (24.7)	30 (30.6)	< 0.0001

*Note:* Values are shown as Median (IQR), or *n* (%). Group 1: No AT indication; Group 2: AT indicated, not treated; Group 3: AT indicated, treated.

Abbreviations: AF, atrial fibrillation; AT, antithrombotic therapy; CKD, chronic kidney disease; HD, heart disease; HS, hemorrhagic stroke; IS, ischemic stroke; SAH, subarachnoid hemorrhages.

^a^ Obesity indicates BMI ≥ 28 kg/m^2^; missing data: 116.

^b^ It did not include hypertension in our study.

^c^ Immediate family member with hemorrhagic stroke.

^d^ It included extracranial bleedings 4 weeks prior to intracerebral hemorrhage.

^e^ It included surgeries 1 month prior to intracerebral hemorrhage.

^f^ Use of antiplatelet agents 2 weeks prior to intracerebral hemorrhage.

^g^ Use of anticoagulants 2 weeks prior to intracerebral hemorrhage.

**TABLE 2:** Clinical characteristics and treatments of patients with intracerebral hemorrhage at baseline.

	Group 1, *N* = 256	Group 2, *N* = 247	Group 3, *N* = 98	*p*
Clinical features				
Symptomatic onset	248 (96.9)	229 (92.7)	81 (82.7)	< 0.0001
In‐hospital onset	38 (14.8)	78 (31.6)	25 (25.5)	< 0.0001
SBP at admission	134 (119–153)	133 (120–148)	132 (116–145)	0.2974
DBP at admission	80 (70–91)	77 (68–86)	77 (66–86)	0.0068
GCS at admission^a^	15 (7–15)	11 (5–15)	15 (7–15)	0.0051
Baseline mRS	4 (2–5)	4 (3–5)	4 (2–5)	0.0001
Baseline ICH‐score	1 (0–2)	1 (0–3)	1 (0–2)	0.0002
Baseline HAS‐BLED	2 (1–3)	3 (2–4)	3 (2–4)	< 0.0001
Radiologic features				
Baseline volume, mL^b^	17.9 (6.1–38.4)	17.0 (4.8–39.0)	1.9 (0.6–12.0)	< 0.0001
Intraventricular	72 (28.1)	77 (31.2)	15 (15.3)	0.0108
Etiology				
Structural vascular lesions	21 (8.2)	13 (5.3)	14 (14.3)	< 0.0001
Medication	7 (2.7)	48 (19.4)	18 (18.4)	
Amyloid angiopathy	15 (5.9)	6 (2.4)	0 (0.0)	
Systemic disease	44 (17.2)	47 (19.0)	12 (12.2)	
Hypertension	84 (32.8)	55 (22.3)	13 (13.3)	
Hemorrhagic transformation	0 (0.0)	16 (6.5)	30 (30.6)	
Intracranial tumor	27 (10.5)	6 (2.4)	0 (0.0)	
Undetermined	58 (22.7)	56 (22.7)	11 (11.2)	
CHA_2_DS_2_‐VASc	NA	3 (2–4)^c^	5 (3–7)^d^	0.0043
Essen Stroke Risk Score	NA	3 (2–4)^e^	3 (1–4)^f^	0.4808
Treated with neurosurgery	115 (44.9)	92 (37.2)	23 (23.5)	0.0009
Treated with craniotomy	57 (22.3)	42 (17.0)	10 (10.2)	0.0259
ICU	90 (35.2)	108 (43.7)	42 (42.9)	0.1185
Days in ICU after onset	4 (2–9)	5 (2–13)	13 (6–33)	< 0.0001
Days from out‐hospital onset to admission	3 (0–13)	2 (0–15)	12 (2–27)	0.0006
Days from admission to in‐hospital onset	5 (1–12)	7 (3–17)	4 (2–11)	0.1454
Length of stay, days	13 (7–21)	13 (6–21)	22 (12–38)	< 0.0001
Follow‐up time	21 (11–36)	20 (7–38)	36 (24–62)	< 0.0001

*Note:* Values are shown as Median (IQR), or *n* (%). Group 1: No AT indication; Group 2: AT indicated, not treated; Group 3: AT indicated, treated.

Abbreviations: AT, antithrombotic therapy; DBP, diastolic blood pressure; GCS, glasgow coma scale; ICU, intensive care unit; mRS, modified Rankin Scale; NA, not applicable; SBP, systolic blood pressure.

^a^ Missing data: 3.

^b^ Missing data were 121.

^c^ 40 patients were included.

^d^ 23 patients were included.

^e^ 107 patients were included.

^f^ 62 patients were included.

### 3.2 Antithrombotic Therapy

Of 345 patients with an AT indication, 98 (28.4%) were restarted on therapy during hospitalization. Antiplatelet agents were used in 42 patients (12.2%), anticoagulants in 55 (15.9%), and both in 1 (0.3%). Median time from onset to AT resumption was 11 days (IQR 2–27). Median time from onset to restart was 7 days (IQR 0–30) for antiplatelets and 14 days (IQR 5–27) for anticoagulants (Tables S2 and S3). The ratio of mono‐ to dual‐antiplatelet therapy was 13:1; the ratio of warfarin: NOAC:heparin/LMWH/argatroban was approximately 3:1:3 (Tables S4 and S5).

### 3.3 Outcome

**FIGURE 1:**
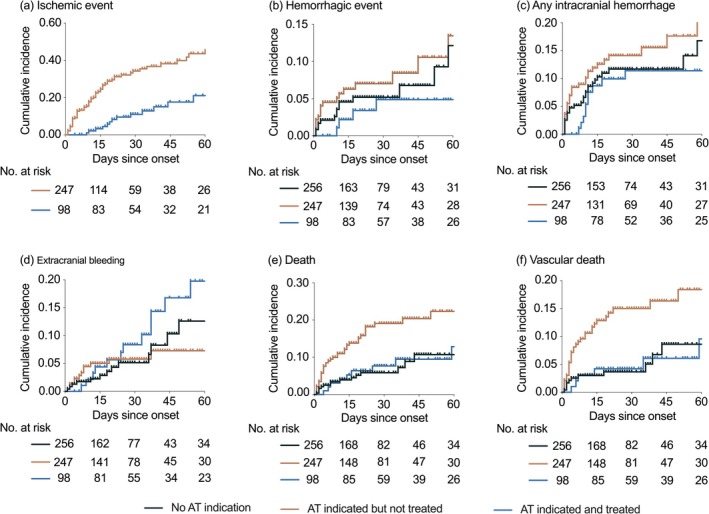
Cumulative incidence of event outcomes in patients with intracerebral hemorrhage by restart of antithrombotic therapy. The tick marks indicate censored participants. Log‐rank tests: (a) *p* < 0.0001; (b) *p* = 0.1038; (c) *p* = 0.0240; (d) *p* = 0.6299; (e) *p* = 0.0013; (f) *p* = 0.0017. AT, antithrombotic therapy.

Overall event rates were ischemic events 17.6% (106), hemorrhagic events 7.5% (45), any intracerebral hemorrhage 15.5% (93), extracranial bleeding 9.3% (56), and all‐cause mortality 11.8% (71) (Table 3 and Table S6). The cumulative incidence of clinical event is shown in Figure 1.

**TABLE 3:** Models for clinical outcomes and treatments in patients with intracerebral hemorrhage.

	Group 1, *N* = 256	Group 2, *N* = 247	Group 3, *N* = 98	*p*
Ischemic event				
Rate	NA	71 (28.7)	14 (14.3)	0.0049
Unadjusted HR	NA	1 [Reference]	0.30 (0.17–0.54)	< 0.0001
Adjusted HR^a^	NA	1 [Reference]	0.34 (0.18–0.65)	0.0011
Hemorrhagic event				
Rate	16 (6.3)	22 (8.9)	5 (5.1)	0.3537
Unadjusted HR	1 [Reference]	1.49 (0.78–2.84)	0.57 (0.21–1.55)	0.1173
Adjusted HR^b^	1 [Reference]	1.69 (0.80–3.56)	0.67 (0.20–2.19)	0.1364
Any intracerebral hemorrhage				
Rate	32 (12.5)	45 (18.2)	11 (11.2)	0.1117
Unadjusted HR	1 [Reference]	1.56 (0.99–2.45)	0.71 (0.36–1.41)	0.0283
Adjusted HR^b^	1 [Reference]	2.04 (1.20–3.47)	0.82 (0.35–1.92)	0.0047
Extracranial bleeding				
Rate	20 (7.8)	18 (7.3)	14 (14.3)	0.0932
Unadjusted HR	1 [Reference]	0.96 (0.51–1.81)	1.32 (0.66–2.62)	0.6331
Adjusted HR^c^	1 [Reference]	0.59 (0.28–1.24)	1.12 (0.43–2.93)	0.1808
mRS ≤ 3 at discharge				
Rate	127 (49.6)	76 (30.8)	47 (48.0)	< 0.0001
Unadjusted OR	1 [Reference]	0.45 (0.31–0.65)	0.94 (0.59–1.49)	0.0010
Adjusted OR^d^	1 [Reference]	0.68 (0.35–1.32)	1.25 (0.53–2.90)	0.2716
GCS ≥ 9 at discharge				
Rate	199 (77.7)	145 (58.7)	78 (79.6)	< 0.0001
Unadjusted OR	1 [Reference]	0.41 (0.28–0.60)	1.12 (0.63–1.98)	< 0.0001
Adjusted OR^e^	1 [Reference]	0.49 (0.28–0.89)	1.15 (0.51–2.62)	0.0191
DAMA or all‐cause death				
Rate	56 (21.9)	87 (35.2)	20 (20.4)	0.0009
Unadjusted HR	1 [Reference]	1.67 (1.19–2.34)	0.73 (0.43–1.21)	0.0003
Adjusted HR^f^	1 [Reference]	1.07 (0.68–1.68)	0.78 (0.41–1.51)	0.5621
All‐cause death				
Rate	20 (7.8)	42 (17.0)	9 (9.2)	0.0041
Unadjusted HR	1 [Reference]	2.28 (1.34–3.88)	0.91 (0.42–2.01)	0.0020
Adjusted HR^f^	1 [Reference]	1.36 (0.66–2.83)	0.84 (0.30–2.37)	0.4634
Vascular death				
Rate	16 (6.3)	35 (14.2)	6 (6.1)	0.0047
Unadjusted HR	1 [Reference]	2.36 (1.31–4.26)	0.78 (0.31–2.00)	0.0027
Adjusted HR^f^	1 [Reference]	1.04 (0.46–2.37)	0.49 (0.14–1.73)	0.3807

*Note:* Values are shown as *n* (%), HR (95% CI), or OR (95% CI). Group 1: No AT indication; Group 2: AT indicated, not treated; Group 3: AT indicated, treated.

Abbreviations: DAMA, discharge against medical advice; GCS, glasgow coma scale; HR, hazard ratio; mRS, modified Rankin Scale; NA, not applicable; OR, odds ratio.

^a^ Adjusted for age, sex, hypertension, diabetes mellitus, dyslipidemia, heart disease, stroke, chronic kidney disease, malignant tumor, smoking, drinking, symptomatic onset, in‐hospital onset, baseline glasgow coma scale, baseline mRS score, baseline ICH‐score, baseline HAS‐BLED score, intraventricular hemorrhage, etiology, and extracranial bleedings 4 weeks prior to intracerebral hemorrhage. 342 patients were included in analysis.

^b^ Adjusted for age, sex, hypertension, diabetes mellitus, thrombocytopenia, stroke, chronic kidney disease, malignant tumor, smoking, drinking, symptomatic onset, in‐hospital onset, baseline glasgow coma scale, baseline mRS score, baseline ICH‐score, baseline HAS‐BLED score, intraventricular hemorrhage, etiology, and extracranial bleedings 4 weeks prior to intracerebral hemorrhage. 598 patients were included in analysis.

^c^ Adjusted for age, sex, hypertension, diabetes mellitus, heart disease, thrombocytopenia, stroke, chronic kidney disease, malignant tumor, smoking, drinking, symptomatic onset, in‐hospital onset, baseline glasgow coma scale, baseline mRS score, baseline ICH‐score, baseline HAS‐BLED score, intraventricular hemorrhage, etiology, and extracranial bleedings 4 weeks prior to intracerebral hemorrhage. 598 patients were included in analysis.

^d^ Adjusted for age, sex, malignant tumors, smoking, drinking, symptomatic onset, in‐hospital onset, baseline glasgow coma scale, baseline mRS score, baseline ICH‐score, baseline HAS‐BLED score, intraventricular hemorrhage, etiology, intensive care unit, transfusion therapy, craniotomy, ischemic event, hemorrhagic event, extracranial bleeding, and comorbid infection. 598 patients were included in analysis.

^e^ Adjusted for age, sex, malignant tumors, smoking, baseline glasgow coma scale, baseline mRS score, baseline ICH‐score, baseline HAS‐BLED score, intraventricular hemorrhage, intensive care unit, transfusion therapy, craniotomy, and hemorrhagic event. 598 patients were included in analysis.

^f^ Adjusted for age, sex, hypertension, diabetes mellitus, heart disease, stroke, chronic kidney disease, malignant tumors, smoking, drinking, symptomatic onset, in‐hospital onset, baseline glasgow coma scale, baseline mRS score, baseline ICH‐score, baseline HAS‐BLED score, intraventricular hemorrhage, etiology, intensive care unit, transfusion therapy, craniotomy, ischemic event, hemorrhagic event, and comorbid infection. 598 patients were included in analysis.

Restarting AT significantly reduced ischemic events compared with patients with an AT indication but no therapy (14.3% vs. 28.7%; adjusted HR 0.34, 95% CI 0.18–0.65). AT resumption was not associated with an increased risk of hemorrhagic events (5.1% vs. 6.3%; adjusted HR 0.67, 95% CI 0.20–2.19). Similarly, the risk of any intracerebral hemorrhage, extracranial bleeding, and mortality did not differ significantly between patients restarted on AT and those without AT indication. In contrast, patients with an AT indication who remained untreated had a higher risk of hematoma expansion or new intracerebral hemorrhage compared with patients without AT indication (18.2% vs. 12.5%; adjusted HR 2.04, 95% CI 1.20–3.47)(Table 3).

### 3.4 Competing Risk Model

Fine–Gray models showed that restarting antithrombotic drugs significantly reduced the risk of ischemic events (adjusted HR = 0.35, 95% CI: 0.19–0.63). In contrast, restarting therapy did not increase the risk of hemorrhagic events, intracerebral hemorrhage, or extracranial bleeding (Table 4).

**TABLE 4:** Fine–Gray hazard models for ischemic and bleeding outcomes.

	Group 1, *N* = 256	Group 2, *N* = 247	Group 3, *N* = 98	*p*
Ischemic event				
Unadjusted HR	NA	1 [Reference]	0.34 (0.20–0.59)	0.0001
Adjusted HR^a^	NA	1 [Reference]	0.35 (0.19–0.63)	0.0005
Hemorrhagic event				
Unadjusted HR	1 [Reference]	1.40 (0.74–2.66)	0.58 (0.22–1.58)	0.1660
Adjusted HR^b^	1 [Reference]	1.75 (0.83–3.67)	0.73 (0.22–2.44)	0.1193
Any intracerebral hemorrhage				
Unadjusted HR	1 [Reference]	1.50 (0.96–2.35)	0.73 (0.37–1.42)	0.0421
Adjusted HR^b^	1 [Reference]	2.09 (1.18–3.70)	0.84 (0.35–2.04)	0.0048
Extracranial bleeding				
Unadjusted HR	1 [Reference]	0.89 (0.47–1.68)	1.34 (0.69–2.59)	0.4658
Adjusted HR^c^	1 [Reference]	0.62 (0.30–1.27)	1.25 (0.52–3.04)	0.2376

*Note:* Group 1: No AT indication; Group 2: AT indicated, not treated; Group 3: AT indicated, treated.

Abbreviations: HR, hazard ratio; NA, not applicable.

^a^ Adjusted for age, sex, hypertension, diabetes mellitus, dyslipidemia, heart disease, stroke, chronic kidney disease, malignant tumor, smoking, drinking, symptomatic onset, in‐hospital onset, baseline glasgow coma scale, baseline mRS score, baseline ICH‐score, baseline HAS‐BLED score, intraventricular hemorrhage, etiology, and extracranial bleedings 4 weeks prior to intracerebral hemorrhage. 342 patients were included in analysis.

^b^ Adjusted for age, sex, hypertension, diabetes mellitus, thrombocytopenia, stroke, chronic kidney disease, malignant tumor, smoking, drinking, symptomatic onset, in‐hospital onset, baseline glasgow coma scale, baseline mRS score, baseline ICH‐score, baseline HAS‐BLED score, intraventricular hemorrhage, etiology, and extracranial bleedings 4 weeks prior to intracerebral hemorrhage. 598 patients were included in analysis.

^c^ Adjusted for age, sex, hypertension, diabetes mellitus, heart disease, thrombocytopenia, stroke, chronic kidney disease, malignant tumor, smoking, drinking, symptomatic onset, in‐hospital onset, baseline glasgow coma scale, baseline mRS score, baseline ICH‐score, baseline HAS‐BLED score, intraventricular hemorrhage, etiology, and extracranial bleedings 4 weeks prior to intracerebral hemorrhage. 598 patients were included in analysis.

### 3.5 Subgroup Analysis

Subgroup analyses demonstrated consistent reductions in ischemic events with antithrombotic therapy across all groups, particularly in patients aged ≥ 50 years or with ICH scores ≤ 3. The HRs for AT in reducing ischemic events were 0.23 (95% CI: 0.11–0.49) in patients admitted within 14 days of onset, and 0.62 (95% CI: 0.24–1.61) in those admitted between 15 and 180 days, with a *p*‐value for interaction of 0.1027. In addition, AT did not increase the hazard ratios of hemorrhagic event in any subgroups with no significant interactions (Table S7).

### 3.6 Sensitivity Analysis

Adjusting for baseline hematoma volume did not change the direction of results (Table S8). Both antiplatelets (adjusted HR 0.25, 95% CI 0.08–0.71) and anticoagulants (adjusted HR 0.18, 95% CI 0.08–0.39) reduced ischemic events versus untreated patients with an AT indication. Antiplatelet use was associated with higher odds of mRS ≤ 3 at discharge (adjusted HR 4.07, 95% CI 1.14–14.49), while anticoagulant use reduced mortality (adjusted HR 0.31, 95% CI 0.11–0.93) (Table S9).

### 3.7 Etiology Analysis of Recurrent Intracerebral Hemorrhage

In patients with restart of antithrombotic drugs, 2 (18.2%) any intracerebral hemorrhages and 2 (40.0%) hemorrhagic events attributed to primary intracerebral hemorrhage, while 9 (81.8%) any intracerebral hemorrhages and 3 (60.0%) hemorrhagic events attributed to restart of antithrombotic drugs (Tables S10 and S11). For those restarting AT, the interval from restart to hemorrhagic events ranged from 4 to 22 days (Table S12).

## 4 Discussion

In this retrospective cohort, restarting antithrombotic therapy (AT) during hospitalization for acute spontaneous ICH was associated with a 66% reduction in subsequent ischemic events without an increased risk of recurrent hemorrhage. This magnitude of benefit was consistent with previous evidence showing that resuming anticoagulation after ICH can reduce ischemic stroke, systemic embolism, and mortality in atrial fibrillation populations [10]. Notably, most previous studies initiated therapy after discharge—77% of anticoagulants were prescribed within 3 months post‐ICH and 74% of antiplatelets beyond 30 days in randomized trials [7]. Our findings address a critical gap by providing direct evidence that in‐hospital, early‐phase resumption of AT can be both feasible and effective.

The clinical need for AT was substantial: 57.4% of patients in our cohort had a clear indication during admission. Yet, only 28.4% of eligible patients were restarted on therapy, a pattern consistent with prior reports of 11%–45% [5, 20]. This discrepancy reflects ongoing clinical hesitation, largely driven by concerns over rebleeding [8, 21, 22]. Interestingly, in our cohort, patients with an AT indication but left untreated exhibited higher rates of hematoma expansion compared to those restarted on therapy. This may reflect selection bias, as early hematoma growth or rebleeding could prompt clinicians to defer AT resumption.

Our results are concordant with a meta‐analysis reporting a pooled relative risk of 0.34 (95% CI 0.25–0.45) for thromboembolic prevention in patients resuming anticoagulants [23]. They also parallel Liu et al.'s recent findings that early antiplatelet resumption after ICH reduced ischemic complications without increasing major bleeding [9]. Conversely, a randomized trial demonstrated no clear vascular benefit of antiplatelets in occlusive disease prevention [7]. Different from Nielsen PB's study, in our study, both Cox and Fine–Gray models confirmed that restarting AT during hospitalization reduced ischemic events, and sensitivity analyses demonstrated consistent benefit for both antiplatelet and anticoagulant therapy [10]. The observation that clinicians preferentially restarted AT in patients perceived as lower‐risk underscores the potential for selection bias and emphasizes the need for randomized validation [24].

A key feature of this study was the inclusion of patients admitted within 6 months of onset, encompassing both acute (≤ 14 days) and subacute (15–180 days) presentations. This design reflected real‐world practice in tertiary centers where severe ICH often necessitates prolonged hospitalization [12]. Subgroup analyses demonstrated similar risk reductions across both windows, with the strongest effect seen in acute‐phase admissions (HR = 0.23), supporting early inpatient resumption in carefully selected patients. The effect of AT resumption between 2 weeks to 6 months appeared to be somewhat diminished, as indicated by a higher hazard ratio (HR = 0.62, 95% CI: 0.24–1.61), suggesting that clinicians should interpret this with caution.

The timing of AT resumption in our study was shorter than in most prior work. Murthy et al. suggested an optimal anticoagulation window of 10–30 weeks, with actual restart times ranging 16–60 days depending on indication and etiology [8, 10, 25, 26, 27]. For antiplatelets, previous studies reported median restart times of 24–229 days [7, 10, 22].

Murthy et al. proposed the optimal time for restart of anticoagulants as 10–30 weeks, but the median time was 16–60 days, varied by indication of AT, etiology of ICH and studies in practice. Researchers debated when to initiate anticoagulants, but generally favored initiating anticoagulants safely after 2 weeks from onset [23, 28]. For antiplatelet therapy, the median restart time ranged from 24 to 229 days, varied by studies. In contrast, our median restart was 4–12 days for antiplatelets and 12–14 days for anticoagulants, with no observed increase in hemorrhagic complications. These findings suggest that a 1–2 weeks inpatient window may be reasonable in appropriately selected patients, though prospective studies are needed to confirm safety [9].

We used patients without AT indication as a control group and demonstrated that resumption of AT was not associated with increased intracerebral or extracranial bleeding, consistent with long‐term findings in prior cohorts [21, 22]. Incorporating this “blank control” provided a baseline estimate of hemorrhagic risk in patients without thromboembolic pressure, thereby reducing indication bias and minimizing confounding from clinicians' risk perception when deciding on AT resumption. This internal reference strengthened the validity of our safety analysis. Although our study was not powered for mortality endpoints, we observed a favorable trend in all‐cause and vascular death, in line with previous work [10, 22].

The regimen of AT initiation reflected standard indications, with minimal use of concomitant anticoagulation and antiplatelet therapy. The warfarin: NOAC ratio (3:1) and mono‐to‐dual antiplatelet ratio (13:1) were comparable to other real‐world cohorts [21, 25, 29], providing insight into practical prescribing patterns during hospitalization.

Predictors of ICH recurrence such as cerebral amyloid angiopathy, microbleed burden, lobar location, and blood pressure control remain critical when considering AT resumption [30, 31, 32]. In patients taking antithrombotic drugs, 81.8% of recurrent hemorrhages were temporally associated with AT restart, whereas 18.2% were attributable to primary ICH progression in our study. This emphasizes the importance of individualized risk assessment, integrating imaging biomarkers and etiology into decision‐making. The observation that more than half of CHIRONE participants restarted vitamin K antagonists after recurrent ICH highlights the ongoing tension between ischemic and hemorrhagic risk [27].

Our study's strengths included a relatively large, well‐characterized ICH cohort with adjudicated outcomes, the use of patients without AT indication as a control to mitigate selection bias, and competing‐risk modeling to account for mortality. The inclusion of a six‐month admission window enhances external validity by reflecting real‐world referral and prolonged inpatient management patterns. Distinct from most prior studies focusing solely on atrial fibrillation, our analysis included both anticoagulant and antiplatelet indications, expanding the applicability of findings beyond a single patient subgroup [8, 10, 30].

## 5 Limitations

Our study has several limitations. First, the retrospective and observational design, coupled with the heterogeneous nature of antithrombotic therapies, introduces inherent challenges. A significant portion of baseline hematoma volume data was missing (approximately 20%). Although we conducted a complete‐case analysis, this may introduce selection bias. A sensitivity analysis excluding patients with missing hematoma volume showed consistent results with the primary findings. Additionally, the lack of systematic analysis of key neuroimaging biomarkers, such as microbleed burden and hematoma morphology, may have limited our ability to fully assess bleeding risk and AT safety. Future studies should aim to incorporate these biomarkers for more refined risk stratification. Second, certain subtypes of ICH, such as lobar ICH and cerebral amyloid angiopathy, were underrepresented, particularly as no patients with amyloid‐related ICH were restarted on AT. This highlights ongoing uncertainty in this subgroup, which warrants further investigation. Third, due to sample size limitations, propensity score matching was not used to mitigate baseline imbalances between groups, which may have exaggerated the effect of AT on reducing ischemic events. Additionally, small sample sizes prevented more granular subgroup analyses of antithrombotic regimens (e.g., warfarin vs. mono/dual antiplatelet therapies). The heterogeneity in these therapies may influence ischemic and hemorrhagic outcomes. Finally, although our study demonstrates statistical associations between AT resumption and clinical outcomes, it does not establish causality. Future prospective, studies with larger sample sizes and longer follow‐up periods are needed to confirm our findings, address the heterogeneity in drug regimens, and incorporate important neuroimaging biomarkers for a more comprehensive understanding of AT resumption after ICH.

## 6 Conclusion

Patients with acute spontaneous ICH face high competing risks of ischemic and hemorrhagic complications. This study demonstrates that restarting AT during hospitalization can substantially reduce ischemic events without increasing recurrent ICH, supporting the clinical value of carefully timed inpatient resumption. These findings provide real‐world evidence to guide individualized therapy and underscore the need for prospective trials to define optimal timing and patient selection.

## Author Contributions

S.L. designed and wrote the manuscript. J.N. and B.P. designed and revised the manuscript. T.Q., X.Z., Q.M., and J.N. gave constructive advice and participated in proofreading of this paper. All authors contributed to the article and approved the submitted version.

## Funding

This study was funded by CAMS Innovation Fund for Medical Sciences (CIFMS) (2022‐I2M‐1‐001) and Clinical Research Information Support Platform (2023‐PUMCH‐F‐002).

## Ethics Statement

This study was approved by the Ethics Committee of Peking Union Medical College Hospital, with a waiver of informed consent (I‐22PJ776).

## Conflicts of Interest

The authors declare no conflicts of interest.

## Supporting information


**Table S1:** Clinical characteristics and treatments of patients with intracerebral hemorrhage at baseline.
**Table S2:** Antithrombotic drugs by types of indication.
**Table S3:** Days from onset to restart of antithrombotic drugs.
**Table S4:** Antiplatelet agents by types of indication.
**Table S5:** Anticoagulants by types of indication.
**Table S6:** Overall rates of clinical event by pre‐ and post‐antithrombotic therapy.
**Table S7:** Subgroup analysis for ischemic and hemorrhagic outcomes.
**Table S8:** Models for clinical outcomes and treatments in patients with intracerebral hemorrhage adjusted for volume of hemorrhagic lesion at baseline.
**Table S9:** Sensitivity analysis for clinical outcomes and treatments with patients with intracerebral hemorrhage and AT indications.
**Table S10:** Any intracerebral hemorrhage after antithrombotic therapy.
**Table S11:** Hemorrhagic event after antithrombotic therapy.
**Table S12:** Days from restart of antithrombotic therapy to hemorrhagic event.
**Figure S1:** Flow chart of Restart‐R cohort.

## Data Availability

The data that support the findings of this study are available from the corresponding author upon reasonable request.
