# Supplementary material for: Antithrombotic Therapy After Intracerebral Hemorrhage: Real‐World Evidence for In‐Hospital Resumption
Source: CNS Neurosci Ther. 2026 Apr 28;32(5):e70883. doi: 10.1002/cns.70883 (PMC13125426; doi:10.1002/cns.70883)
Supplement: Supplementary file 1 — Table S1: Clinical characteristics and treatments of patients with intracerebral hemorrhage at baseline. Table S2: Antithrombotic drugs by types of indication. Table S3: Days from onset to restart of antithrombotic drugs. Table S4: Antiplatelet agents by types of indication. Table S5: Anticoagulants by types of indication. Table S6: Overall rates of clinical event by pre‐ and post‐antithrombotic therapy. Table S7: Subgroup analysis for ischemic and hemorrhagic outcomes. Table S8: Models for clinical outcomes and treatments in patients with intracerebral hemorrhage adjusted for volume of hemorrhagic lesion at baseline. Table S9: Sensitivity analysis for clinical outcomes and treatments with patients with intracerebral hemorrhage and AT indications. Table S10: Any intracerebral hemorrhage after antithrombotic therapy. Table S11: Hemorrhagic event after antithrombotic therapy. Table S12: Days from restart of antithrombotic therapy to hemorrhagic event. Figure S1: Flow chart of Restart‐R cohort. [file CNS-32-e70883-s001.docx]

**Supplementary material**

**Table S1. Clinical characteristics and treatments of patients with intracerebral hemorrhage at baseline**

|  | Group 1  N=256 | Group 2  N=247 | Group 3  N=98 | P value |
| --- | --- | --- | --- | --- |
| Laboratory features |  |  |  |  |
| Hemoglobin, g/L | 130 (109-147) | 119 (91-138) | 122 (90-140) | 0.0005 |
| Platelet, 10^9^/L | 203 (148-246) | 167 (98-234) | 191 (139-266) | 0.0025 |
| Platelet< 100*10^9^/L | 44 (17.2) | 62 (25.1) | 13 (13.3) | 0.0174 |
| Creatinine, μmol/L | 68 (56-82) | 72 (55-100) | 72 (55-96) | 0.0244 |
| INR ≥ 1.5 | 9 (3.5) | 19 (7.7) | 8 (8.2) | 0.0873 |
| Radiologic features |  |  |  |  |
| Supratentorial | 201 (78.5) | 202 (81.8) | 85 (86.7) | 0.1990 |
| Subtentorial | 28 (10.9) | 28 (11.3) | 11 (11.2) | 0.9896 |
| Extradural | 0 (0.0) | 1 (0.4) | 0 (0.0) | 0.4878 |
| Subdural | 3 (1.2) | 5 (2.0) | 2 (2.0) | 0.7189 |
| SAH | 18 (7.0) | 18 (7.3) | 7 (7.1) | 0.9938 |
| Lobe | 111 (43.4) | 108 (43.7) | 55 (56.1) | 0.0727 |
| Non-lobe | 96 (37.5) | 96 (38.9) | 33 (33.7) | 0.6675 |
| Indication of antiplatelet therapy | NA | 160 (64.8) | 76 (77.6) | 0.0214 |
| Ischemic stroke | NA | 107 (43.3) | 62 (63.3) | 0.0008 |
| Coronary heart disease | NA | 53 (21.5) | 19 (19.4) | 0.6697 |
| Others | NA | 34 (13.8) | 14 (14.3) | 0.8997 |
| Indication of anticoagulant therapy | NA | 161 (65.2) | 82 (83.7) | 0.0007 |
| Atrial fibrillation | NA | 40 (16.2) | 23 (23.5) | 0.1147 |
| Coronary artery disease | NA | 12 (4.9) | 7 (7.1) | 0.4015 |
| Valvular heart disease | NA | 7 (2.8) | 11 (11.2) | 0.0016 |
| DVT/PE | NA | 89 (36.0) | 49 (50.0) | 0.0169 |
| Others | NA | 50 (20.2) | 22 (22.4) | 0.6493 |
| With transfusion therapy | 51 (19.9) | 77 (31.2) | 29 (29.6) | 0.0112 |
| Comorbid infection | 70 (27.3) | 100 (40.5) | 34 (34.7) | 0.0078 |

DVT, deep vein thrombosis; NA, not applicable; PE, pulmonary embolism;

**Table S2. Antithrombotic drugs by types of indication**

|  | Indication of antiplatelet therapy | Indication of anticoagulant therapy | Indication of antiplatelet and anticoagulant therapy | P value |
| --- | --- | --- | --- | --- |
| Types of drugs |  |  |  | <0.0001 |
| None | 86 (84.3) | 87 (79.8) | 74 (55.2) |  |
| Antiplatelet agents | 16 (15.7) | 0 (0) | 26 (19.4) |  |
| Anticoagulants | 0 (0) | 22 (20.2) | 33 (24.6) |  |
| Both | 0 (0) | 0 (0) | 1 (0.8) |  |

Values are shown as n (%).

**Table S3. Days from onset to restart of antithrombotic drugs**

|  | Indication of antiplatelet therapy | Indication of anticoagulant therapy | Indication of antiplatelet and anticoagulant therapy |
| --- | --- | --- | --- |
| Types of drugs |  |  |  |
| Antiplatelet agents | 12 (1-56) | … | 4 (0-16) |
| Anticoagulants | … | 12 (4-26) | 14 (6-32) |
| Both | … | … | 12 (12-12) |

Values are shown as n (%).

**Table S4. Antiplatelet agents by types of indication**

|  | Indication of antiplatelet therapy | Indication of anticoagulant therapy | Indication of antiplatelet and anticoagulant therapy | P value |
| --- | --- | --- | --- | --- |
| Types of antiplatelet agents |  |  |  | < 0.0001 |
| None | 86 (84.3) | 109 (100.0) | 107 (79.9) |  |
| Mono | 15 (14.7) | 0 (0) | 25 (18.7) |  |
| Dual | 1 (1.0) | 0 (0) | 2 (1.5) |  |

Values are shown as n (%).

**Table S5. Anticoagulants by types of indication**

|  | Indication of anti-platelet therapy | Indication of anti-coagulant therapy | Indication of antiplatelet and antico-agulant therapy | P value |
| --- | --- | --- | --- | --- |
| Types of anticoagulants |  |  |  | < 0.0001 |
| None | 102 (100) | 87 (78.6) | 100 (74.6) |  |
| Warfarin | 0 (0) | 1 (0.9) | 1 (0.8) |  |
| Heparin/LMWH sequential warfarin | 0 (0) | 12 (11.0) | 12 (9.0) |  |
| NOAC | 0 (0) | 1 (0.9) | 2 (1.5) |  |
| Heparin/LMWH sequential NOAC | 0 (0) | 1 (0.9) | 4 (3.0) |  |
| Pure heparin/LMWH/argatroban | 0 (0) | 7 (6.4) | 15 (11.2) |  |

Values are shown as n (%).

LMWH, low molecular weight heparin; NOAC, novel oral anticoagulants.

**Table S6. Overall rates of clinical event by pre- and post-antithrombotic therapy**

|  | Total  N=601 | Group 1  N=256 | Group 2  N=247 | Group 3  N=98 | P value |
| --- | --- | --- | --- | --- | --- |
| Ischemic event after onset |  |  |  |  | <0.0001 |
| No | 495 (82.4) | 256 (100) | 176 (71.3) | 63 (64.3) |  |
| Before AT | 21 (3.5) | 0 (0) | 0 (0) | 21 (21.4) |  |
| After AT | 85 (14.1) | 0 (0) | 71 (28.7) | 14 (14.3) |  |
| Hemorrhagic event after onset |  |  |  |  | 0.0153 |
| No | 556 (92.5) | 240 (93.8) | 225 (91.1) | 91 (92.9) |  |
| Before AT | 2 (0.3) | 0 (0) | 0 (0) | 2 (2.0) |  |
| After AT | 43 (7.2) | 16 (6.3) | 22 (8.9) | 5 (5.1) |  |
| Any intracerebral hemorrhage after onset |  |  |  |  | <0.0001 |
| No | 508 (84.5) | 224 (87.5) | 202 (81.8) | 82 (83.7) |  |
| Before AT | 5 (0.8) | 0 (0) | 0 (0) | 5 (5.1) |  |
| After AT | 88 (14.6) | 32 (12.5) | 45 (18.2) | 11 (11.2) |  |
| Extracranial bleeding after onset |  |  |  |  | <0.0001 |
| No | 545 (90.7) | 236 (92.2) | 229 (92.7) | 80 (81.6) |  |
| Before AT | 4 (0.7) | 0 (0) | 0 (0) | 4 (4.1) |  |
| After AT | 52 (8.7) | 20 (7.8) | 18 (7.3) | 14 (14.3) |  |

Values are shown as n (%).

AT, antithrombotic therapy.

Group 1: No AT indication; Group 2: AT indicated, not treated; Group 3: AT indicated, treated.

**Table S7 Subgroup analysis for ischemic and hemorrhagic outcomes**

|  | Group 1  N=256 | Group 2  N=247 | Group 3  N=98 | P value for interaction |
| --- | --- | --- | --- | --- |
| **Ischemic event** |  |  |  |  |
| Age |  |  |  | 0.1308 |
| ≤ 49y | NA | 1 [Reference] | 0.55 (0.19-1.55) |  |
| ≥ 50y | NA | 1 [Reference] | 0.24 (0.11-0.50) |  |
| Sex |  |  |  | 0.2104 |
| Male | NA | 1 [Reference] | 0.42 (0.20-0.87) |  |
| Female | NA | 1 [Reference] | 0.20 (0.08-0.52) |  |
| ICH score |  |  |  | 0.2259 |
| ≤ 3 | NA | 1 [Reference] | 0.28 (0.15-0.52) |  |
| ≥ 4 | NA | 1 [Reference] | 0.82 (0.16-4.10) |  |
| HAS-BLED score |  |  |  | 0.2331 |
| ≤ 2 | NA | 1 [Reference] | 0.21 (0.08-0.54) |  |
| ≥ 3 | NA | 1 [Reference] | 0.41 (0.20-0.85) |  |
| Etiology |  |  |  | 0.1703 |
| Hypertension | NA | 1 [Reference] | 0.09 (0.01-0.67) |  |
| Non-hypertension | NA | 1 [Reference] | 0.39 (0.21-0.71) |  |
| Days from onset to admission |  |  |  | 0.1027 |
| ≤ 14 | NA | 1 [Reference] | 0.23 (0.11-0.49) |  |
| 15-180 | NA | 1 [Reference] | 0.62 (0.24-1.61) |  |
| **Hemorrhagic event** |  |  |  |  |
| Age |  |  |  | 0.0756 |
| ≤ 49y | 1 [Reference] | 3.27 (1.14-9.36) | 1.89 (0.55-6.47) |  |
| ≥ 50y | 1 [Reference] | 1.13 (0.49-2.58) | 0.13 (0.02-1.07) |  |
| Sex |  |  |  | 0.5248 |
| Male | 1 [Reference] | 1.58 (0.67-3.76) | 0.88 (0.27-2.86) |  |
| Female | 1 [Reference] | 1.38 (0.53-3.64) | 0.23 (0.03-1.86) |  |
| ICH score |  |  |  | 0.9999 |
| ≤ 3 | 1 [Reference] | 1.66 (0.86-3.20) | 0.64 (0.23-1.75) |  |
| ≥ 4 | 1 [Reference] | NE | NE |  |
| HAS-BLED score |  |  |  | 0.4536 |
| ≤ 2 | 1 [Reference] | 1.15 (0.45-2.93) | 0.78 (0.22-2.77) |  |
| ≥ 3 | 1 [Reference] | 1.82 (0.60-5.48) | 0.41 (0.08-2.30) |  |
| Etiology |  |  |  | 0.9690 |
| Hypertension | 1 [Reference] | 1.11 (0.25-4.97) | NE |  |
| Non-hypertension | 1 [Reference] | 1.47 (0.71-3.03) | 0.58 (0.20-1.66) |  |
| Days from onset to admission |  |  |  | 0.8924 |
| ≤ 14 | 1 [Reference] | 1.46 (0.67-3.17) | 0.64 (0.18-2.29) |  |
| 15-180 | 1 [Reference] | 1.82 (0.58-5.73) | 0.63 (0.12-3.24) |  |

Values are shown as n (%), HR (95% CI), or OR (95% CI).

HR, hazard ratio; NA, not applicable; NE, not estimable; OR, odds ratio.

Group 1: No AT indication; Group 2: AT indicated, not treated; Group 3: AT indicated, treated.

**Table S8. Models for clinical outcomes and treatments in patients with intracerebral hemorrhage adjusted for volume of hemorrhagic lesion at baseline**

|  | Group 1  N=256 | Group 2  N=247 | Group 3  N=98 | P value |
| --- | --- | --- | --- | --- |
| Ischemic event, HR^a^ | NA | 1 [Reference] | 0.20 (0.09-0.45) | <0.0001 |
| Hemorrhagic event,HR^b^ | 1 [Reference] | 1.86 (0.77-4.49) | 0.76 (0.21-2.75) | 0.1887 |
| Any intracerebral hemorrhage, HR ^b^ | 1 [Reference] | 2.18 (1.20-3.98) | 0.91 (0.36-2.29) | 0.0105 |
| Extracranial bleeding, HR^c^ | 1 [Reference] | 0.64 (0.26-1.55) | 1.50 (0.52-4.38) | 0.2262 |
| mRS ≤ 3 at discharge, OR^d^ | 1 [Reference] | 0.76 (0.36-1.60) | 0.78 (0.29-2.08) | 0.7554 |
| GCS ≥ 9 at discharge, OR^e^ | 1 [Reference] | 0.60 (0.32-1.12) | 1.63 (0.58-4.56) | 0.0657 |
| DAMA or all-cause death, HR^f^ | 1 [Reference] | 1.02 (0.62-1.67) | 0.72 (0.33-1.53) | 0.5959 |
| All-cause death, HR^f^ | 1 [Reference] | 1.24 (0.57-2.70) | 0.33 (0.08-1.31) | 0.1216 |
| Vascular death, HR^f^ | 1 [Reference] | 1.09 (0.46-2.59) | 0.13 (0.02-0.82) | 0.0514 |

Values are shown HR (95% CI), or OR (95% CI).

HR, hazard ratio; NA, not applicable; OR, odds ratio.

Group 1: No AT indication; Group 2: AT indicated, not treated; Group 3: AT indicated, treated.

a Adjusted for age, sex, hypertension, diabetes mellitus, dyslipidemia, heart disease, stroke, chronic kidney disease, malignant tumor, smoking, drinking, symptomatic onset, in-hospital onset, baseline glasgow coma scale, baseline mRS score, baseline ICH score, baseline HAS-BLED score, intraventricular hemorrhage, etiology, extracranial bleedings 4 weeks prior to intracerebral hemorrhage, and hemorrhagic lesion at baseline. 273 patients were included in analysis.

b Adjusted for age, sex, hypertension, diabetes mellitus, thrombocytopenia, stroke, chronic kidney disease, malignant tumor, smoking, drinking, symptomatic onset, in-hospital onset, baseline glasgow coma scale, baseline mRS score, baseline ICH-score, baseline HAS-BLED score, intraventricular hemorrhage, etiology, extracranial bleedings 4 weeks prior to intracerebral hemorrhage, and hemorrhagic lesion at baseline. 477 patients were included in analysis.

c Adjusted for age, sex, hypertension, diabetes mellitus, heart disease, thrombocytopenia, stroke, chronic kidney disease, malignant tumor, smoking, drinking, symptomatic onset, in-hospital onset, baseline glasgow coma scale, baseline mRS score, baseline ICH-score, baseline HAS-BLED score, intraventricular hemorrhage, etiology, extracranial bleedings 4 weeks prior to intracerebral hemorrhage, and hemorrhagic lesion at baseline. 477 patients were included in analysis.

d Adjusted for age, sex, malignant tumors, smoking, drinking, symptomatic onset, in-hospital onset, baseline glasgow coma scale, baseline mRS score, baseline ICH-score, baseline HAS-BLED score, intraventricular hemorrhage, etiology, intensive care unit, transfusion therapy, craniotomy, ischemic event, hemorrhagic event, extracranial bleeding, comorbid infection, and hemorrhagic lesion at baseline. 477 patients were included in analysis.

e Adjusted for age, sex, malignant tumors, smoking, baseline glasgow coma scale, baseline mRS score, baseline ICH-score, baseline HAS-BLED score, intraventricular hemorrhage, intensive care unit, transfusion therapy, craniotomy, hemorrhagic event, and hemorrhagic lesion at baseline. 477 patients were included in analysis.

f Adjusted for age, sex, hypertension, diabetes mellitus, heart disease, stroke, chronic kidney disease, malignant tumors, smoking, drinking, symptomatic onset, in-hospital onset, baseline glasgow coma scale, baseline mRS score, baseline ICH-score, baseline HAS-BLED score, intraventricular hemorrhage, etiology, intensive care unit, transfusion therapy, craniotomy, ischemic event, hemorrhagic event, comorbid infection, and hemorrhagic lesion at baseline. 477 patients were included in analysis.

**Table S9 Sensitivity analysis for clinical outcomes and treatments with patients with intracerebral hemorrhage and AT indications.**

|  | Group 2  N=247 | Group 3 | | P value |
| --- | --- | --- | --- | --- |
|  |  | Antiplatelet agents  N=42 | Anticoagulants  N=56 |  |
| Ischemic event |  |  |  |  |
| Rate | 71 (28.7) | 5 (11.9) | 9 (16.1) | 0.0172 |
| Unadjusted HR | 1 [Reference] | 0.25 (0.10-0.63) | 0.34 (0.17-0.68) | 0.0003 |
| Adjusted HR^a^ | 1 [Reference] | 0.25 (0.08-0.71) | 0.18 (0.08-0.39) | <0.0001 |
| Hemorrhagic event |  |  |  |  |
| Rate | 22 (8.9) | 2 (4.8) | 3 (5.4) | 0.4917 |
| Unadjusted HR | 1 [Reference] | 0.35 (0.08-1.52) | 0.42 (0.12-1.40) | 0.1658 |
| Adjusted HR^b^ | 1 [Reference] | 0.25 (0.05-1.27) | 0.21 (0.05-0.88) | 0.0477 |
| Any intracerebral hemorrhage |  |  |  |  |
| Rate | 45 (18.2) | 5 (11.9) | 6 (10.7) | 0.2795 |
| Unadjusted HR | 1 [Reference] | 0.50 (0.20-1.26) | 0.44 (0.19-1.02) | 0.0735 |
| Adjusted HR^b^ | 1 [Reference] | 0.31 (0.11-0.88) | 0.22 (0.08-0.58) | 0.0031 |
| Extracranial bleeding |  |  |  |  |
| Rate | 18 (7.3) | 3 (7.1) | 11 (19.6) | 0.0140 |
| Unadjusted HR | 1 [Reference] | 0.68 (0.20-2.31) | 1.85 (0.87-3.93) | 0.1643 |
| Adjusted HR^c^ | 1 [Reference] | 0.61 (0.15-2.42) | 1.16 (0.47-2.88) | 0.6564 |
| mRS ≤ 3 |  |  |  |  |
| Rate | 76 (30.8) | 25 (59.5) | 22 (39.3) | 0.0013 |
| Unadjusted OR | 1 [Reference] | 3.31 (1.69-6.48) | 1.46 (0.80-2.65) | 0.0019 |
| Adjusted OR^d^ | 1 [Reference] | 4.07 (1.14-14.49) | 0.92 (0.26-3.26) | 0.0739 |
| All-cause death |  |  |  |  |
| Rate | 42 (17.0) | 4 (9.5) | 5 (8.9) | 0.1815 |
| Unadjusted HR | 1 [Reference] | 0.43 (0.15-1.19) | 0.37 (0.15-0.95) | 0.0418 |
| Adjusted HR^e^ | 1 [Reference] | 0.40 (0.10-1.58) | 0.31 (0.11-0.93) | 0.0821 |
| Vascular death |  |  |  |  |
| Rate | 35 (14.2) | 3 (7.1) | 3 (5.4) | 0.1101 |
| Unadjusted HR | 1 [Reference] | 0.39 (0.12-1.27) | 0.28 (0.09-0.92) | 0.0413 |
| Adjusted HR^e^ | 1 [Reference] | 0.32 (0.06-1.67) | 0.26 (0.07-0.99) | 0.0882 |

Values are shown HR (95% CI), or OR (95% CI).

HR, hazard ratio; OR, odds ratio.

Group 2: AT indicated, not treated; Group 3: AT indicated, treated. As shown in eTable 1, one patient received both antiplatelet agents and anticoagulants, they are classed into subgroup of anticoagulants (N=56).

a Adjusted for age, sex, hypertension, diabetes mellitus, dyslipidemia, stroke, heart disease, chronic kidney disease, smoking, drinking, symptomatic onset, in-hospital onset, baseline glasgow coma scale, baseline mRS score, baseline ICH-score, baseline HAS-BLED score, intraventricular hemorrhage, etiology, extracranial bleedings 4 weeks prior to intracerebral hemorrhage, and types of AT indication. 342 patients were included in analysis.

b Adjusted for age, sex, hypertension, heart disease, thrombocytopenia, stroke, drinking, baseline glasgow coma scale, baseline mRS score, baseline ICH-score, baseline HAS-BLED score, intraventricular hemorrhage, etiology, extracranial bleedings 4 weeks prior to intracerebral hemorrhage, and types of AT indication. 342 patients were included in analysis.

c Adjusted for age, sex, heart disease, thrombocytopenia, autoimmune disease, stroke, baseline glasgow coma scale, baseline mRS score, baseline ICH-score, baseline HAS-BLED score, intraventricular hemorrhage, etiology, extracranial bleedings 4 weeks prior to intracerebral hemorrhage and types of AT indication. 342 patients were included in analysis.

d Adjusted for age, sex, malignant tumors, smoking, drinking, symptomatic onset, in-hospital onset, baseline glasgow coma scale, baseline mRS score, baseline ICH-score, baseline HAS-BLED score, intraventricular hemorrhage, etiology, intensive care unit, transfusion therapy, craniotomy, ischemic event, hemorrhagic event, and types of AT indication. 342 patients were included in analysis.

e Adjusted for age, sex, heart disease, stroke, malignant tumors, smoking, symptomatic onset, in-hospital onset, baseline glasgow coma scale, baseline mRS score, baseline ICH-score, baseline HAS-BLED score, intraventricular hemorrhage, etiology, intensive care unit, transfusion therapy, craniotomy, ischemic event, hemorrhagic event, comorbid infection and types of AT indication. 342 patients were included in analysis.

**Table S10. Any intracerebral hemorrhage after antithrombotic therapy**

|  | Total  N=88 | Group 1  N=32 | Group 2  N=45 | Group 3  N=11 | P value |
| --- | --- | --- | --- | --- | --- |
| Reasons |  |  |  |  | < 0.0001 |
| Antithrombotic therapy | 10 (11.4) | 0 (0) | 1 (2.2) | 9 (81.8) |  |
| Primary intracerebral hemorrhage | 75 (85.2) | 29 (90.6) | 44 (97.8) | 2 (18.2) |  |
| Others | 3 (3.4) | 3 (9.4) | 0 (0) | 0 (0) |  |

Group 1: No AT indication; Group 2: AT indicated, not treated; Group 3: AT indicated, treated.

One patient suffered from any intracerebral hemorrhage due to “antithrombotic therapy”, as this event might be caused by the antithrombotic therapy prior to primary intracerebral hemorrhage.

**Table S11. Hemorrhagic event after antithrombotic therapy**

|  | Total  N=43 | Group 1  N=16 | Group 2  N=22 | Group 3  N=5 | P value |
| --- | --- | --- | --- | --- | --- |
| Reasons |  |  |  |  | <0.0001 |
| Antithrombotic therapy | 4 (9.3) | 0 (0) | 1 (4.6) | 3 (60.0) |  |
| Primary intracerebral hemorrhage | 37 (86.1) | 14 (87.5) | 21 (95.4) | 2 (40.0) |  |
| Others | 2 (4.7) | 2 (12.5) | 0 (0) | 0 (0) |  |

Group 1: No AT indication; Group 2: AT indicated, not treated; Group 3: AT indicated, treated. One patient suffered from hemorrhagic event due to “antithrombotic therapy”, as hemorrhagic event might be caused by the antithrombotic therapy prior to primary intracerebral hemorrhage.

**Table S12. Days from restart of antithrombotic therapy to hemorrhagic event**

| No. | Age/sex | Type of AT drug | Interval, days | Reasons |
| --- | --- | --- | --- | --- |
| 1 | M/39 | Anticoagulants | 4 | Antithrombotic therapy |
| 2 | M/79 | Antiplatelet agents | 22 | Antithrombotic therapy |
| 3 | F/42 | Antiplatelet agents | 6 | Antithrombotic therapy |
| 4 | M/49 | Anticoagulants | 8 | Primary intracerebral hemorrhage |
| 5 | M/29 | Anticoagulants | 6 | Primary intracerebral hemorrhage |

**Figure S1 Flow chart of Restart-R cohort**

Group 3: AT indicated, treated. (N= 98)

Group 2: AT indicated, not treated (N= 247)

Group 1: No AT indication (N= 256)

Excluded (N=394)

- Traumatic ICH (N= 369)
- Pure cerebral microbleeds (N= 25)

Spontaneous intracranial hemorrhage (N = 2237)

Patients aged ≥18 years with intracranial hemorrhage hospitalized in Peking Union Medical College Hospital between November 2014 and October 2022 (N= 2631)

Excluded (N= 1636)

- Related to neurosurgery (N= 41)
- Time from onset to admission > 6 months (N= 1489)
- Pure SAH, extradural or subdural hemorrhage (N=106)

Acute spontaneous intracerebral hemorrhage (N = 601)

AT, antithrombotic therapy. Group 1: No AT indication; Group 2: AT indicated, not treated; Group 3: AT indicated, treated.
